# Potential role of transthoracic echocardiography for screening LV systolic dysfunction in patients with a history of dengue infection. A cross-sectional and cohort study and review of the literature

**DOI:** 10.1371/journal.pone.0276725

**Published:** 2022-11-18

**Authors:** Molly D. Kaagaard, Alma Wegener, Laura C. Gomes, Anna E. Holm, Karine O. Lima, Luan O. Matos, Isabelle V. M. Vieira, Rodrigo Medeiros de Souza, Lasse S. Vestergaard, Claudio Romero Farias Marinho, Flávia Barreto Dos Santos, Tor Biering-Sørensen, Odilson M. Silvestre, Philip Brainin

**Affiliations:** 1 Multidisciplinary Center, Federal University of Acre, Cruzeiro do Sul, Acre, Brazil; 2 Department of Cardiology, Copenhagen University Hospital–Herlev and Gentofte, Herlev, Denmark; 3 Department of Parasitology, Institute of Biomedical Sciences, University of São Paulo, São Paulo, Brazil; 4 National Malaria Reference Laboratory, Department of Bacteria, Parasites and Fungi, Statens Serum Institut, Copenhagen, Denmark; 5 Laboratório de Imunologia Viral, Instituto Oswaldo Cruz, Fiocruz, Rio de Janeiro, RJ, Brazil; 6 Faculty of Biomedical Sciences, Copenhagen University, Copenhagen, Denmark; 7 Health and Sport Science Center, Federal University of Acre, Rio Branco, Acre, Brazil; Charité Universitätsmedizin Berlin - Campus Virchow-Klinikum: Charite Universitatsmedizin Berlin - Campus Virchow-Klinikum, GERMANY

## Abstract

**Background:**

Dengue virus can affect the cardiovascular system and men may be at higher risk of severe complications than women. We hypothesized that clinical dengue virus (DENV) infection could induce myocardial alterations of the left ventricle (LV) and that these changes could be detected by transthoracic echocardiography.

**Methodology/Principal findings:**

We examined individuals from Acre in the Amazon Basin of Brazil in 2020 as part of the Malaria Heart Study. By questionnaires we collected information on self-reported prior dengue infection. All individuals underwent transthoracic echocardiography, analysis of left ventricular ejection fraction (LVEF) and global longitudinal strain (GLS). We included 521 persons (mean age 40±15 years, 39% men, 50% urban areas) of which 253 (49%) had a history of dengue infection. In multivariable models adjusted for clinical and sociodemographic data, a history of self-reported dengue was significantly associated with lower LVEF (β = -2.37, P < 0.01) and lower GLS (β = 1.08, P < 0.01) in men, whereas no significant associations were found in women (P > 0.05). In line with these findings, men with a history of dengue had higher rates of LV systolic dysfunction (LVEF < 50% = 20%; GLS < 16% = 17%) than those without a history of dengue (LVEF < 50% = 7%; GLS < 16% = 8%; P < 0.01 and 0.06, respectively).

**Conclusions/Significance:**

The findings of this study suggest that a clinical infection by dengue virus could induce myocardial alterations, mainly in men and in the LV, which could be detected by conventional transthoracic echocardiography. Hence, these results highlight a potential role of echocardiography for screening LV dysfunction in participants with a history of dengue infection. Further larger studies are warranted to validate the findings of this study.

## Introduction

Dengue fever affects approximately 390 million people worldwide each year and the prevalence is rising [1]. Dengue is a viral vector-borne disease transmitted primarily by the mosquito *Aedes aegypti*, which is common in urban areas [2]. It belongs to the genus Flavivirus and has four distinct serotypes. Consequently, persons may be infected up to four times and repeated infection is typically associated with worse clinical outcome [2]. The clinical presentation ranges from non-specific symptoms to severe courses with shock and respiratory distress resulting from plasma leakage, severe bleeding, or severe organ impairment (2009 classification by the World Health Organization) [2]. Diagnosis of dengue in the early phase is traditionally performed using polymerase chain reaction or rapid diagnostic tests for non-structural antigen 1 (NS1). However, in areas with sparse access to laboratory tests, physician diagnosis based on clinical signs and symptoms is also common. Later, diagnosis is made serologically by detecting IgM and IgG. Dengue may be confused with other arboviruses such as Zika and Chikungunya, as all three diseases may have unspecific symptom, and antibodies against each virus can cross-react. However, the amount of Zika cases has decreased drastically since the initial epidemic, and in 2019 there were 691,000 confirmed cases of dengue and 1,800 confirmed cases of Zika in Brazil [3].

It has been proposed that dengue virus (DENV) can affect individual organ systems, including the cardiovascular system, where it has been associated with myocarditis, electrical abnormalities [4] and left ventricular (LV) impairment [5,6]. While these complications have been observed in the acute phase of the infection, no studies have assessed the long-term effect on the heart following recovery from dengue. Recently, studies have proposed that men may be at higher risk of severe dengue complications, especially cardiovascular complications [7,8]. Therefore, the primary aim of this study was to evaluate whether a history of clinical dengue relates to lower cardiac function, more specifically in men. To address this, we applied speckle tracking echocardiography, an imaging technique which can detect even subtle changes in the contractile function of the heart. We hypothesized that a history of clinical dengue was associated with worsening in LV function in individuals from Amazon Basin of Brazil, and that this relationship was more pronounced in men. Our secondary aim was to assess sociodemographic characteristics associated with DENV infection.

## Methods

### Study site

**Fig 1:**
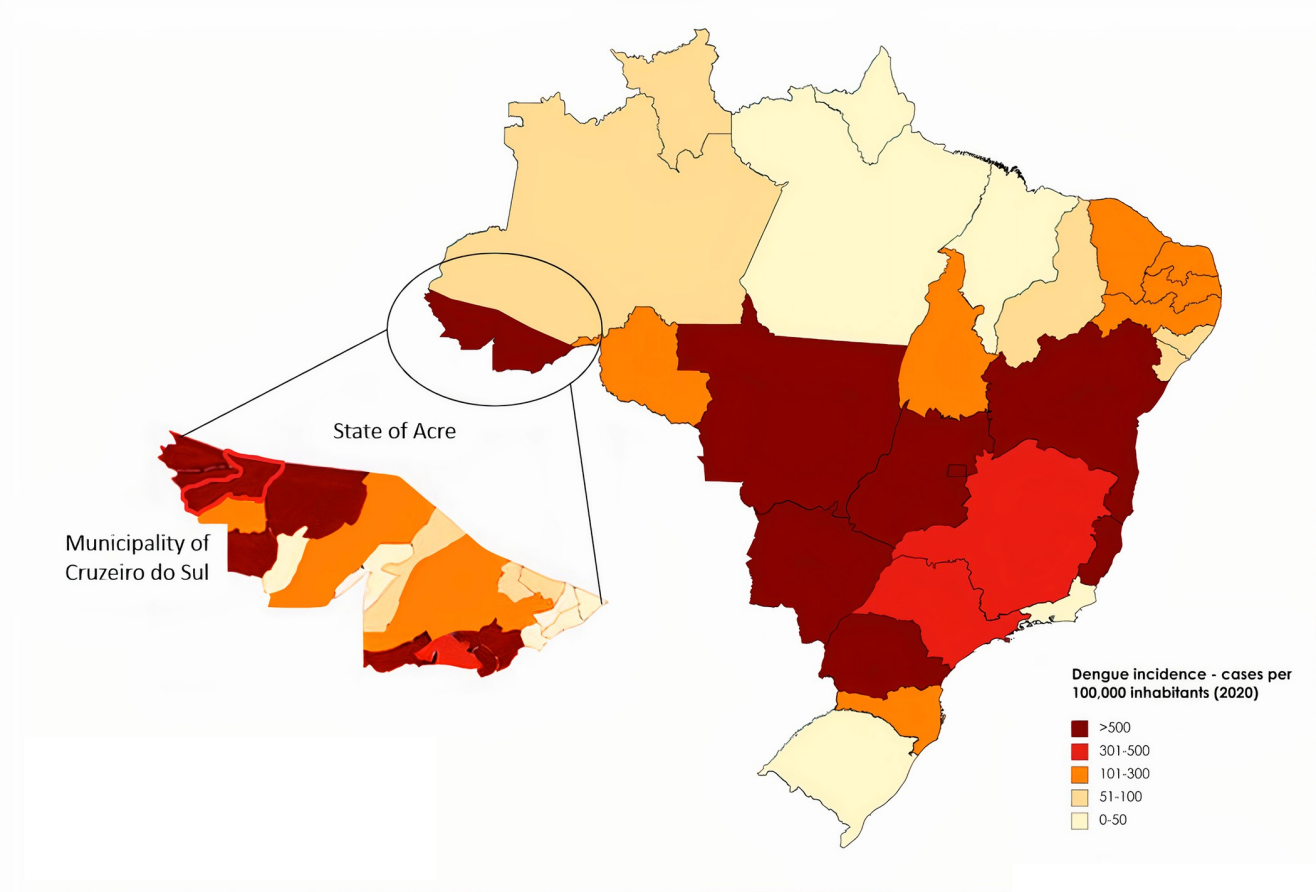
Dengue incidence in Brazil. Map of dengue incidence in Brazil in week 1 to 26, 2020. The municipality of Cruzeiro do Sul, within the state of Acre, is marked with a red line. The map was reprinted with permission from www.MapChart.net under the CC BY 4.0 license. Data on dengue incidence is from a report by Ministerio da Saúde Brazil [9,13].

The study was conducted in the municipality of Cruzeiro do Sul (8,816 km^2^), Acre, Western part of the Brazilian Amazon. In 2020, Acre had 7,986 confirmed dengue cases (906 cases/100,000 inhabitants), which is above the average in Brazil (470 cases/100,000 inhabitants) (Fig 1). Both DENV-1 and DENV-2 appear in Acre, of which DENV-2 is considered predominant [9]. Dengue has been endemic in Acre since the early 2000’s (S1 Table) [10]. However, all four serotypes circulate in Brazil [9]. In recent years urbanization of Cruzeiro do Sul has increased significantly [11]. In addition to this, the area is well-known for a relatively high incidence of malaria infections and has an annual parasite index of >10 [12].

### Study population

**Fig 2:**
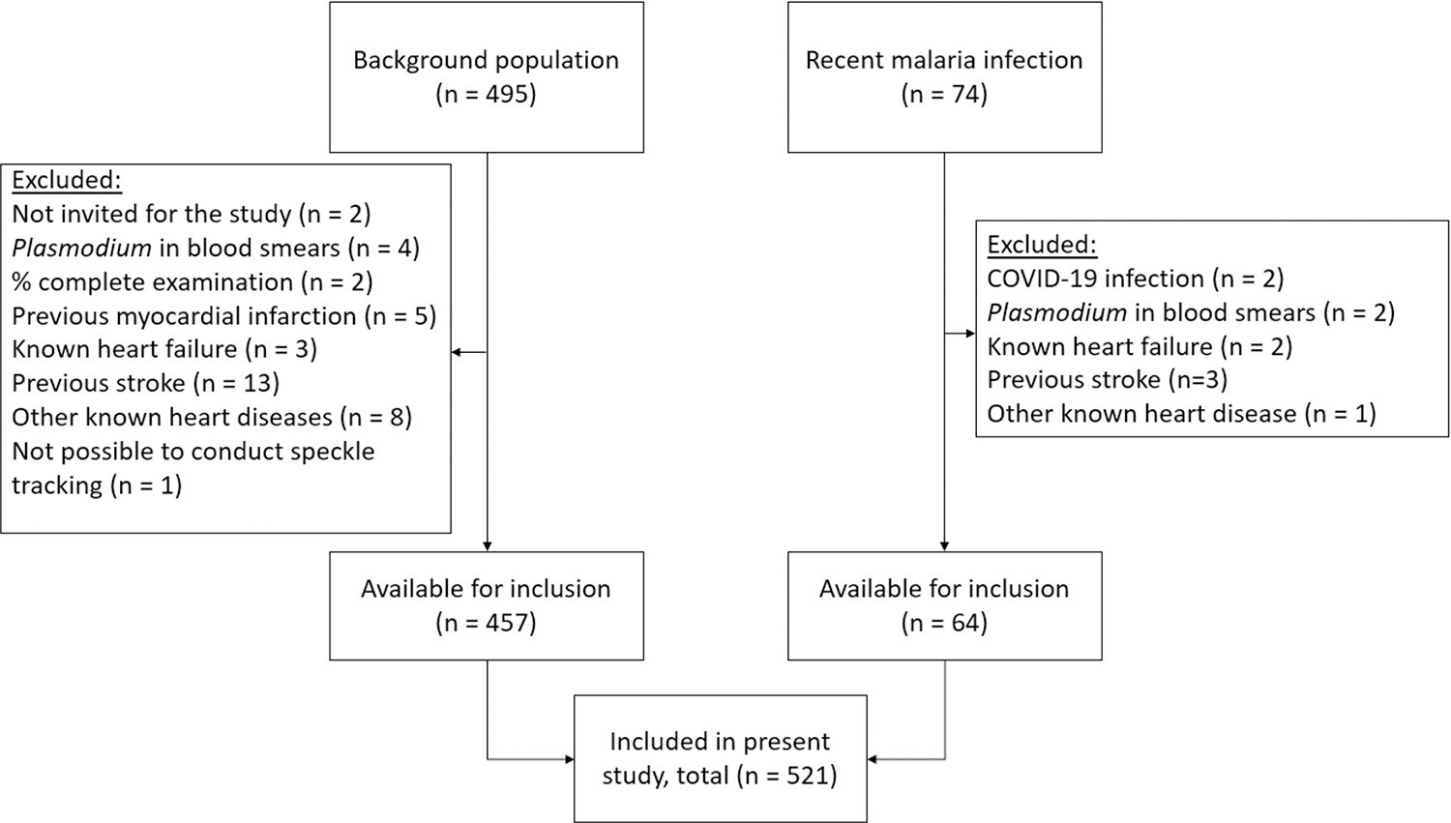
Flowchart of inclusion. Overview of reasons for exclusion of study participants.

This was a cross-sectional, observational cohort study, conducted as a part of the Malaria Heart Study, which included participants from June 2020 to December 2020 (clinicaltrials.gov: NCT04445103). We enrolled participants from 10 local healthcare clinics in the municipality of Cruzeiro do Sul, Acre, equally distributed between urban (n = 5) and rural areas (n = 5). This was done due to socioeconomic differences between urban and rural residents adhering to the main protocol. Local healthcare agents provided lists of persons pertaining to each clinic, from which a random sample was invited. The specific inclusion criteria for this secondary study were age >18 years, knowledge about prior dengue episode(s) (either confirmed by a diagnostic test or physician diagnosis) throughout the participant’s life, and completion of the examination program. All participants in the Malaria Heart Study fulfilled these criteria. We excluded participants with any suspected ongoing infection determined by a medical doctor, presence of *Plasmodium* in peripheral blood smears, referral from the examination site to a cardiologist because of suspected cardiac disease, prior myocardial infarction or stroke, heart failure, known pregnancy and missing speckle tracking data (Fig 2). Furthermore, we included a second group of participants, who recently had completed anti-malarial treatment (median 31 days ago). All participants from this group fulfilled the inclusion and exclusion criteria described above and had negative peripheral blood smears (Fig 2).

### Data collection

We performed interviewed questionnaires in Portuguese to obtain information on self-reported clinical dengue, prior cardiovascular disease and risk factors, cardiovascular symptoms including shortness of breath and intermittent claudication, current medication and sociodemographic characteristics (income, work, education, type of house). Clinical dengue was defined as symptoms of dengue (fever, headache, retroorbital pain, exanthema, prostration, myalgia, arthralgia) combined with a positive dengue test or physician diagnosis. Diagnostic tests for COVID-19 were not readily available during the study period. Instead, participants with a relevant history of recent symptoms (fever and cough) and self-reported prior COVID-19 infection were categorized as suspected history of COVID-19. Participants underwent a physical examination with measurement of height, weight, abdominal circumference, assessment of lower extremity edema and recording of electrocardiograms (ECG). A physician (PB) examined all participants for signs of ongoing infectious disease. All ECGs were analyzed for LV hypertrophy, left and right bundle branch block and presence of pathological Q-waves. Additional details on data collection, classification of symptoms, ECG analyses and laboratory procedures are available in (S1 Appendix).

### Laboratory analyses

A random subset of the population (n = 40), equally distributed by history/no history of clinical dengue, underwent antibody analyses of DENV IgG. A positive history of dengue was defined as elevated IgG above a locally accepted reference limit applied by the laboratory (Citolab, Cruzeiro do Sul, Acre). Analyses were done by immunofluorescence test of serum (specificity 99%, sensitivity 98% according to the manufacturer; ECO Reader F100, ECO Diagnóstica, Brazil), which is sensitive to DENV-1 to DENV-4 [14].

### Conventional echocardiography

A single expert investigator (PB) performed bedside echocardiography (Vivid IQ, GE Healthcare, Horten, Norway). Examinations were analyzed offline in EchoPac (GE Vingmed, BT13, v.203.82). One investigator (AW), blinded to clinical data and dengue status, analyzed conventional parameters according to guidelines [15]. LVEF was assessed by Simpson’s biplane method in the apical two- and four-chamber views. LV end diastolic dimensions were measured in the parasternal long axis view at the level of the mitral valve leaflet tips, and LV mass index (LVMI) was calculated by the Devereux formula [16]. Left atrial volumes were obtained using the area-length method in apical two- and four-chamber views in end-systole and diastole. Accordingly, we calculated the left atrial volume index (LAVI). Peak early (E) and late (A) mitral inflow velocities and deceleration time of the E-wave were measured in the apical four chamber view using pulsed-wave Doppler with the sample placed at the tip of the mitral valve leaflets. Peak early diastolic myocardial velocity (e’) was measured in the same view by tissue Doppler imaging with the pulsed-wave sample placed above the lateral and septal mitral annulus, and the E/e’ ratio was calculated. Tricuspid annular plane systolic excursion (TAPSE) was measured in M-mode through the lateral tricuspid annulus in the apical four-chamber view. Rheumatic heart disease was assessed according to criteria from World Heart Federation [17].

### Speckle tracking echocardiography

Blinded to all data, an experienced investigator (MK) conducted the speckle tracking analyses according to guidelines [18]. The mean frame rate was 55±5 frames/second. A region of interest was defined in the apical two, three and four chamber views by placing three samples, one in the apex and two at the base of the LV. In the parasternal short axis view we placed four samples, equally distributed along the endocardium. The tracking was visually evaluated and accepted if it covered the entire wall from endocardium to the epicardial border, and motion of speckles was visible. When necessary, the region of interest was manually readjusted. If the tracking remained inadequate, the segment in question was excluded. Six myocardial wall segments (septal, lateral, anterior, posterior, anteroseptal and inferior) were examined, yielding a total of 18 segments. Global longitudinal strain (GLS) was calculated as the average of peak global strain values from all segments. Global circumferential strain (GCS) was calculated as an average of strain from the papillary and apical levels. GLS was available in all participants (100%) whereas GCS was available in 433 participants (82%). As both GLS and GCS represent shortening of myocardial fibers, their values are negative, and an increase represents decreasing myocardial function. Furthermore, we assigned each segment one point if it had a score >-16%, and then summarized these scores for each participant, yielding a score from 0 to 18, consequently reflecting the number of segments with hypokinesia.

### Ethics

The Malaria Heart Study was approved by the institutional review committees at Federal University of Acre and University of São Paulo (CAAE: 26552619.6.0000.510 and 32947520.4.0000.5467), local health care authorities and leaders of health care clinics. The study complies with the 2^nd^ Declaration of Helsinki, and all participants provided written informed consent after having received oral and written information about the study in Portuguese. Illiterate participants provided finger prints on consent forms, which was verified by two independent witnesses. For ethical reasons, a physician was always present during examination of participants.

### Statistics

Two-sided P-values <0.05 were considered significant. Distribution of continuous variables were assessed by histograms and Q-Q plots and income was log-transformed to a normal distribution. Baseline characteristics were stratified according to self-reported history of dengue. Categorical variables were compared using Pearson’s chi-squared test, normally distributed variables by Student’s t-test and skewed variables by Wilcoxon rank-sum test. Kruskal-Wallis test was used for comparison of three or more groups. We examined the relationship between number of ECG alterations and echocardiographic abnormalities using a linear regression model. Cardiac symptoms and clinical findings across groups with altered LVEF and/or GLS were compared using Pearson’s chi-squared test. The relationships between dengue and echocardiographic variables were analyzed in linear regression models, and based on our a priori hypothesis, we assessed men and women. Multivariable models included relevant confounders and variables from Table 1: Age, systolic blood pressure, heart rate, income, creatinine, smoking, diabetes, rural/urban area, body mass index (BMI) and recent malaria infection. The relationship between LVEF, GLS, GCS and dengue was displayed in logistic spline models. Number of knots were determined according to the lowest Akaike information criterion. The relationship between sociodemographic variables and dengue was assessed by stepwise forward logistic regression. A P<0.10 was the criterion for covariates to enter the multivariable model. No variables displayed collinearity, defined as variance inflation factor <5 (S2 Table). Sensitivity and specificity of self-reported dengue to predict seropositivity were calculated using the *diagt* command in STATA. Statistical analyses were performed in STATA IC version 13.1 (StataCorp LP, College Station, TX).

**Table 1:** Baseline characteristics stratified by history of dengue.

	No history of dengue (n = 268)	History of dengue (n = 253)	P
**Baseline**			
Age, years	39 ± 15	40 ± 14	0.63
Men, n(%)	121 (45%)	84 (33%)	0.005
Recent malaria infection, n(%)	29 (11%)	35 (14%)	0.30
BMI, kg/m^2^	26 ± 5	28 ± 5	0.009
Present smoker, n(%)	102 (38%)	84 (33%)	0.25
Hypertension, n(%)	85 (32%)	100 (40%)	0.063
Hypercholesterolemia, n%)	38 (14%)	39 (15%)	0.99
Diabetes, n(%)	12 (5%)	15 (6%)	0.46
SBP, mmHg	130 ± 19	132 ± 19	0.26
Heart rate, bpm	71.4 ± 12	75.9 ± 13	<0.001
Rheumatic heart disease, n(%)	9 (3%)	3 (1%)	0.099
History of COVID-19, n(%)	17 (6%)	29 (12%)	0.040
Number of dengue episodes			NA
1	NA	165 (65%)	
2	NA	61 (24%)	
3–4	NA	27 (11%)	
**Socioeconomic**			
Family income in real, BRL	1,150 (800 to 2,000)	1,500 (1,000 to 2,500)	0.003
Family income in Euros, €	219 (152 to 380)	285 (190 to 475)	
Insecure job situation, n(%)	168 (63%)	136 (54%)	0.039
Education, n(%)			0.15
No formal education	20 (8%)	11 (4%)	
Primary school	96 (36%)	85 (34%)	
Secondary school	113 (42%)	104 (41%)	
Higher academic	39 (15%)	53 (21%)	
Urban living area, n(%)	101 (38%)	159 (63%)	<0.001
House type, n(%)			0.002
Wood	199 (74%)	156 (62%)	
Brick	69 (26%)	97 (38%)	
Use of mosquito bed net, n(%)	153 (57%)	129 (51%)	0.16
Use of mosquito repellent, n(%)	10 (8%)	20 (8%)	0.85
**Biochemistry**			
CRP, mg/dL	0.0 (0.0 to 0.0)	0.0 (0.0 to 0.0)	0.51
Hemoglobin, g/dL	14.2 ± 1.3	14.0 ± 1.4	0.085
Leukocytes, mm^3^	6100 (5000 to 7360)	6360 (5310 to 7690)	0.078
Reticulocytes, %	0.8 (0.6 to 0.9)	0.8 (0.6 to 0.9)	0.77
Platelets, mm^3^	234 ± 77	236 ± 64	0.74
Creatinine, mg/dL	0.8 (0.7 to 1.0)	0.9 (0.7 to 1.0)	0.010
Bilirubin total, mg/dL	0.4 (0.2 to 0.5)	0.3 (0.2 to 0.5)	0.034
INR	1.03 ± 0.11	1.00 ± 0.10	0.001
Blood glucose, mg/dL	94 (86 to 109)	96 (87 to 115)	0.059
**Electrocardiogram**			
Left ventricular hypertrophy, n(%)	20 (8%)	10 (4%)	0.086
Left bundle branch block, n(%)	0 (0%)	0 (0%)	NA
Right bundle branch block, n(%)	0 (0%)	2 (1%)	0.14
Pathological Q-waves, n(%)	7 (3%)	2 (1%)	0.11
**Echocardiography**			
LV ejection fraction, %	58 ± 5	57 ± 5	0.48
LVEF<50%, n(%)	13 (5%)	24 (10%)	0.040
GLS, %	-19.5 ± 2	-19.4 ± 2	0.36
GCS, %	-21.1 ± 4	-20.8 ± 4	0.41
GLS>-16%, n(%)	10 (4%)	16 (6%)	0.17
Number of hypokinetic segments	3 (1 to 5)	2 (1 to 5)	0.98
LV mass index, g/m^2^	68.8 ± 16	67.5 ± 17	0.36
LAVI, mL/m^2^	19.6 ± 5	18.4 ± 4	0.001
LAVI>34 mL/m^2^, n(%)	6 (2%)	6 (2%)	0.92
e’, cm/s	13.1 ± 4	13.0 ± 4	0.80
Lateral e’<10 cm/s, n(%)	36 (13%)	38 (15%)	0.60
Septal e’<7 cm/s, n(%)	20 (8%)	27 (11%)	0.20
E/e’>14, n(%)	14 (5%)	10 (4%)	0.49
E/A-ratio	1.3 ± 0.5	1.3 ± 0.4	0.22
TAPSE, mm	2.0 ± 0.3	2.0 ± 0.3	0.57
Tricuspid regurgitation >3.8 m/s, n(%)	1 (<1%)	0 (0%)	0.33

BMI = body mass index, BRL = Brazilian real (local currency), GCS = global circumferential strain, GLS = global longitudinal strain, LAVI = left atrial volume index, LV = left ventricular, LVEF = left ventricular ejection fraction, LVMI = left ventricular mass index, SBP = systolic blood pressure, TAPSE = Tricuspid annular plane systolic excursion.

## Results

We included 521 participants (mean age 40 ± 15 years, 205 (39%) men) of which 253 (49%) reported a history of clinical DENV infection. Persons with a prior episode of dengue were more often women, had higher income, BMI, creatinine and more frequently lived in urban areas in brick houses (P<0.05; Table 1). Mean values of echocardiographic parameters were within normal ranges in both groups. When stratified by sex, there was no difference in the prevalence of diabetes, hypertension, hypercholesterolemia or smoking (P>0.05; S3 Table). Men more frequently had a history of alcohol intake (P<0.05). For comparison, we also looked at baseline values by history of malaria. Participants with a history of malaria were more often men, smokers, had higher hemoglobin, lower platelets, higher LV mass index, and higher LAVI (S4, S5 and S6 Tables). No significant difference was found in LV contractility.

### Dengue and echocardiography

#### Entire population

Individuals with a self-reported history of dengue had lower LAVI (18.4 vs 19.6 mL/m^2^, P = 0.001) compared to no dengue. No differences were observed in other echocardiographic parameters (Table 1). Participants with altered LVEF and/or GLS did not display more cardiovascular symptoms compared to those with normal LV function (Table 2). In adjusted linear regression models, a history of dengue was not associated with LVEF (β = -0.32 (95%CI -1.26 to 0.62), P = 0.50) or GLS (β = 0.21 (95%CI -0.16 to 0.58), P = 0.27), or with other echocardiographic parameters (Table 3).

**Table 2:** Cardiovascular symptoms stratified by altered LVEF and/or GLS.

	LVEF<50% and/or GLS >-16% n = 52	LVEF>50% n = 469	P-value
NYHA ≥ II	3 (6%)	51 (11%)	0.25
Intermittent claudication	7 (14%)	56 (12%)	0.75
Lower extremity edema at clinical examination	25 (5%)	3 (6%)	0.90

GLS = global longitudinal strain, LVEF = left ventricular ejection fraction, NYHA = New York Heart Association functional classification.

**Table 3:** Unadjusted associations between self-reported clinical DENV infection and echocardiographic parameters by linear regression models.

	Entire cohort (n = 521)		Men (n = 205)		Women (n = 316)	
	Beta (95%CI)	P	Beta (95%CI)	P	Beta (95%CI)	P
**Unadjusted**						
GLS	0.16 (-0.19 to 0.51)	0.36	1.15 (0.67 to 1.64)	<0.001*	-0.33 (-0.71 to 0.05)	0.091
GCS	0.32 (-0.43 to 1.07)	0.41	1.44 (0.39 to 2.50)	0.007*	-0.27 (-1.10 to 0.57)	0.53
LVEF	-0.31 (-1.17 to 0.56)	0.48	-2.43 (-3.64 to -1.23)	<0.001*	0.75 (-0.20 to 1.70)	0.12
LVMI	-1.29 (-4.07 to 1.49)	0.36	7.20 (3.36 to 11.04)	<0.001*	-5.51 (-8.53 to -2.49)	<0.001*
LAVI	-1.28 (-2.07 to -0.49)	0.001	-0.28 (-1.40 to 0.83)	0.62	-1.78 (-2.66 to -0.90)	<0.001*
E’	-0.08 (-0.72 to 0.55)	0.80	-0.46 (-1.37 to 0.44)	0.32	0.11 (-0.60 to 0.82)	0.77
E/A ratio	-0.05 (-0.13 to 0.03)	0.22	-0.06 (-0.17 to 0.05)	0.28	-0.04 (-0.13 to 0.05)	0.35
TAPSE	-0.02 (-0.07 to 0.04)	0.57	0.01 (-0.07 to 0.09)	0.83	-0.03 (-0.09 to 0.03)	0.38

*Significant in multivariable models adjusted for age, systolic blood pressure, heart rate, creatinine, diabetes, smoking, body mass index, recent malaria infection, urban/rural living area and income.

GCS = global circumferential strain, GLS = global longitudinal strain, LAVI = left atrial volume index, LVEF = left ventricular ejection fraction, LVMI = left ventricular mass index, SBP = systolic blood pressure, TAPSE = Tricuspid annular plane systolic excursion.

### Men

**Fig 3:**
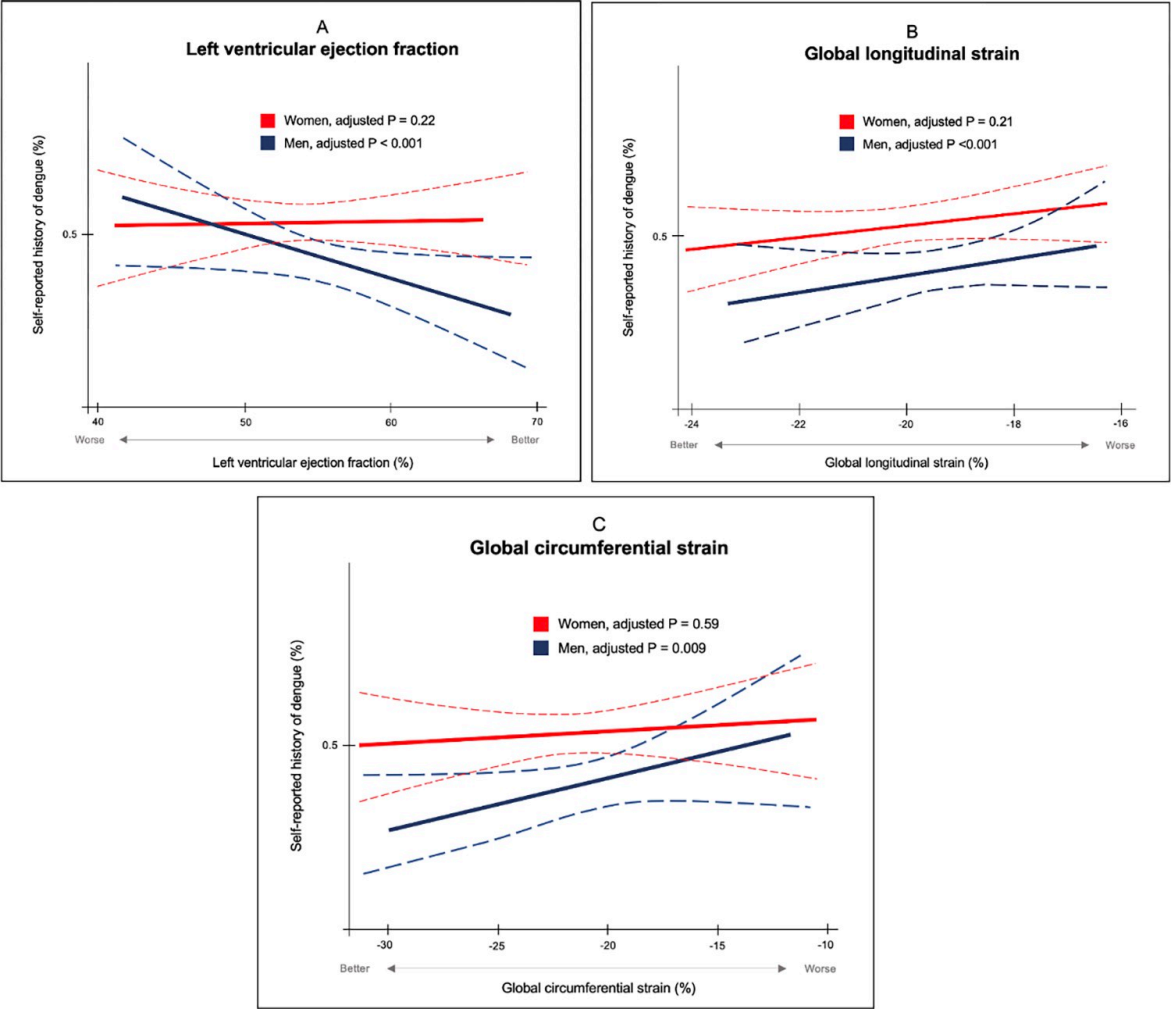
Relationship between myocardial function and dengue. Logistic spline models, stratified by sex, displaying the relationship between parameters of myocardial function (A: Left ventricular ejection fraction, B: Global longitudinal strain, C: Global circumferential strain) and a history of self-reported clinical DENV infection.

Baseline data is displayed in Table 4. LVEF was worse in men with a history of dengue (55% vs. 57%, P = 0.031). Although non-significant, individuals with a self-reported history of dengue also had lower GLS and GCS. Unadjusted associations are shown in Table 3. In multivariable models, a history of dengue was associated with lower LVEF (β = -2.37 (95%CI -3.69 to -1.04), P<0.001), GLS (β = 1.08 (95%CI 0.55 to 1.61), P<0.001) and GCS (β = 1.55 (95%CI 0.38 to 2.71), P = 0.009) (Fig 3A–3C). Dengue was also associated with higher LVMI (β = 5.07 (95%CI 1.55 to 8.60), P = 0.005).

**Table 4:** Baseline characteristics of men stratified by history of clinical DENV infection.

	No history of dengue (n = 121)	History of dengue (n = 84)	P
**Baseline**			
Age, years	38 ± 15	39 ± 14	0.76
Recent malaria infection, n(%)	15 (12%)	21 (25%)	0.020
BMI, kg/m^2^	25 ± 4	27 ± 4	<0.001
Present smoker, n(%)	48 (40%)	34 (41%)	0.91
Hypertension, n(%)	37 (31%)	40 (48%)	0.013
Hypercholesterolemia, n(%)	14 (12%)	9 (11%)	0.85
Diabetes, n(%)	3 (3%)	5 (6%)	0.21
SBP, mmHg	131 ± 16	136 ± 16	0.024
Heart rate, bpm	65 ± 10	72 ± 13	<0.001
Rheumatic heart disease, n(%)	6 (5%)	2 (2%)	0.35
Suspected history of COVID-19, n(%)	7 (6%)	11 (13%)	0.069
Number of dengue episodes			NA
1	NA	55 (66%)	
2	NA	17 (20%)	
3–4	NA	12 (14%)	
**Socioeconomic**			
Family income in real, BRL	1500 (1000 to 2300)	2000 (1000 to 3000)	0.070
Family income in Euros, €	285 (190 to 438)	381 (190 to 571)	
Insecure job situation, n(%)	72 (60%)	38 (45%)	0.044
Education, n(%)			0.23
No formal education	12 (10%)	3 (4%)	
Primary school	43 (36%)	30 (36%)	
Secondary school	49 (41%)	33 (39%)	
Higher academic	17 (14%)	18 (21%)	
Urban living area, n(%)	35 (29%)	54 (64%)	<0.001
House type, n(%)			0.032
Wood	93 (77%)	53 (63%)	
Brick	28 (23%)	31 (37%)	
Use of mosquito bed net, n(%)	59 (49%)	42 (50%)	0.86
Use of mosquito repellent, n(%)	5 (4%)	7 (8%)	0.21
**Biochemistry**			
CRP, mg/dL	0.0 (0.0 to 0.0)	0.0 (0.0 to 0.0)	0.22
Hemoglobin, g/dL	15.2 ± 1	15.3 ± 1	0.38
Leukocytes, mm^3^	5710 (4800 to 6780)	6120 (5170 to 7335)	0.047
Reticulocytes, %	0.8 (0.6 to 0.9)	0.8 (0.6 to 1.0)	0.069
Platelets, mm^3^	221 ± 93	216 ± 59	0.62
Creatinine, mg/dL	0.9 (0.8 to 1.1)	1.0 (0.9 to 1.1)	0.001
Bilirubin total, mg/dL	0.4 (0.3 to 0.6)	0.38 (0.29 to 0.58)	0.093
INR	1.05 ± 0.1	1.01 ± 0.1	0.001
Blood glucose, mg/dL	93 (85 to 108)	96 (87 to 115)	0.18
**Electrocardiogram**			
Left ventricular hypertrophy, n(%)	16 (13%)	8 (10%)	0.42
Left bundle branch block, n(%)	0 (0%)	0 (0%)	NA
Right bundle branch block, n(%)	0 (0%)	1 (1%)	0.23
Pathological Q-waves, n(%)	4 (3%)	1 (1%)	0.33
**Echocardiography**			
LV ejection fraction, %	57 ± 5	55 ± 6	0.031
LVEF<50%, n(%)	9 (7%)	17 (20%)	0.007
GLS, %	-18.8 ± 2	-18.4 ± 2	0.12
GCS, %	-20.6 ± 4	-19.7 ± 4	0.12
GLS>-16%, n(%)	10 (8%)	14 (17%)	0.066
Number of hypokinetic segments	2 (2 to 4)	2 (2 to 5)	0.076
LV mass index, g/m^2^	76 ± 16	76 ± 16	0.96
LAVI, mL/m^2^	21 ± 5	19 ± 5	0.018
LAVI>34 mL/m^2^, n(%)	2 (2%)	2 (2%)	0.71
e’, cm/s	13.6 ± 3.6	12.7 ± 3.4	0.062
Lateral e’<10 cm/s, n(%)	14 (12%)	15 (18%)	0.20
Septal e’<7 cm/s, n(%)	8 (7%)	9 (11%)	0.29
E/e’>14, n(%)	6 (5%)	2 (2%)	0.35
E/A-ratio	1.4 ± 0.5	1.3 ± 0.4	0.055
TAPSE, mm	2.1 ± 0.3	2.0 ± 0.3	0.49
Tricuspid regurgitation >3.8 m/s, n(%)	1 (<1%)	0 (0%)	0.40

BMI = body mass index, BRL = Brazilian real (local currency), GCS = global circumferential strain, GLS = global longitudinal strain, LAVI = left atrial volume index, LV = left ventricular, LVEF = left ventricular ejection fraction, LVMI = left ventricular mass index, SBP = systolic blood pressure, TAPSE = Tricuspid annular plane systolic excursion.

#### Women

No significant differences were observed in parameters of LV function when stratifying women by a history of dengue (S7 Table). Unadjusted associations are displayed in Table 3. In multivariable models, no associations were found with LVEF (β = 0.65 (95%CI -0.38 to 1.67), P = 0.22), GLS (β = -0.21 (95%CI -0.62 to 0.21), P = 0.32) or GCS (β = -0.25 (95%CI -1.15 to 0.65), P = 0.59). However, a history of dengue was significantly associated with lower LVMI (β = -3.88 (95%CI -6.59 to -1.16), P = 0.005) and LAVI (β = -1.01 (95%CI -1.81 to -0.21), P = 0.013).

#### Electrocardiogram

In participants with no dengue history, 20 (8%) had LV hypertrophy and 7 (3%) pathological Q-waves. In participants with prior dengue, 10 (4%) had LV hypertrophy, 2 (1%) right bundle branch block and 2 (1%) pathological Q-waves. None had left bundle branch block. We found no significant difference between the two groups (P>0.05 for all). In the group with prior dengue, most participants had no ECG or echocardiographic abnormalities (Table 5). However, number of ECG alterations increased with number of abnormal echocardiographic findings (P = 0.001).

**Table 5:** ECG alterations versus echocardiographic abnormalities in participants with a history of dengue (n = 253).

	Echocardiographic abnormalities				
ECG alterations	No abnormalities	1 abnormality	2 abnormalities	3 abnormalities	Total
No alterations	201	33	5	1	240
1 alteration	6	5	0	1	12
2 alterations	0	0	1	0	1
3 alterations	0	0	0	0	0
Total	207	38	6	2	253

ECG alterations involve LVH, LBBB, RBBB, pathological Q-waves.

Echocardiographic abnormalities involve LVEF<50%, GLS>-16%, LAVI>34mL/m^2^, E/e’>14.

ECG = electrocardiography, GLS = global longitudinal strain, LAVI = left atrial volume index, LBBB = left bundle branch block, LVEF = left ventricular ejection fraction, LVH = left ventricular hypertrophy, RBBB = right bundle branch block.

*Multiple DENV infections*. When assessing the entire cohort and women, number of self-reported DENV infections (per 1 increase) was not associated with LVEF, GLS or GCS (P>0.05; S8 Table). However, a higher number of DENV infections among men (per 1 increase) was associated with lower LVEF (β = -0.91 (95%CI -1.63 to -0.19), P = 0.013), GLS (β = 0.53 (95%CI 0.24 to 0.82), P<0.001) and GCS (β = 0.86 (95%CI 0.22 to 1.51), P = 0.009) in adjusted models.

### Characteristics associated with dengue

In univariable models, female sex, urban living area, living in a brick house, income and BMI were associated with increased odds of DENV infection. In multivariable analyses, female sex (OR 1.49 (95%CI 1.01–2.19), P = 0.043) and urban living area (OR 2.50 (95%CI 1.65–3.77), P<0.001) were the only parameters that persisted to be significantly associated with DENV infection (Table 6).

**Table 6:** Association between sociodemographic variables and previous DENV infection.

Sociodemographic variable	Unadjusted OR (95% CI)	P	Adjusted OR (95% CI)*	P
**Age**	1.00 (0.99–1.02)	0.54		
**Female sex**	1.66 (1.16–2.36)	0.005	1.49 (1.01–2.19)	0.043
**Urban living area**	2.80 (1.96–3.99)	<0.001	2.50 (1.65–3.77)	<0.001
**Brick house**	1.79 (1.23–2.60)	0.002	1.17 (0.76–1.80)	0.48
**Log(income)**	1.35 (1.09–1.66)	0.005	1.10 (0.84–1.44)	0.49
**Education**	1.25 (1.01–1.54)	0.040	0.94 (0.73–1.21)	0.61
**Employment status**	0.69 (0.49–0.98)	0.039	0.89 (0.59–1.34)	0.58
**Body mass index**	1.05 (1.01–1.08)	0.009	1.04 (1.00–1.08)	0.053
**Use of mosquito bed net**	0.78 (0.55–1.10)	0.16		
**Use of mosquito repellent**	1.06 (0.56–2.03)	0.85		

*In the adjusted analysis, each variable was mutually adjusted for other covariates: Sex, rural/urban area, house type, income, education, employment status, body mass index, mosquito prophylaxis (bed net, repellent).

### Antibody analysis

Fifteen of 20 participants with self-reported history of clinical dengue and 7/20 with no dengue history had a positive antibody titer. Self-reported DENV infection had a sensitivity of 71% (95%CI 48% to 89%) and a specificity of 74% (95%CI 49% to 91%) to predict seropositivity. The positive and negative predictive values of self-reported dengue was 75% and 70%, respectively.

## Discussion

The principal finding in this study is that a history of clinical DENV infection was associated with more impaired myocardial function in men from the Amazon Basin. When assessing the entire population and women, a history of dengue had no relationship with LV myocardial function.

Only few studies with heterogenous populations have examined dengue and cardiovascular complications, and they applied varying definitions of cardiovascular disease [4]. Moreover, a majority of studies have focused on hospitalized participants with severe dengue [4]. Two studies demonstrated that participants with severe dengue often had impaired LV systolic function [5,6], and a study by Kirawittaya et al found diastolic dysfunction during dengue [19]. Notably, the studies had a limited sample size (ranging from 20 to 181 cases) and enrolled participants with uncomplicated dengue as controls for severe dengue cases [6,19]. Following medical care and fluid therapy, cardiac function normalized after three to eight days in most studies. As an exception, a study by Yadav et al [20] examined cardiac function following discharge for dengue infection. A total of 11/67 children with severe dengue continued to have LVEF<50%, indicating that dengue may have a long-term effect on cardiac contractility. Cardiovascular manifestations of dengue during the acute phase as described by various studies are displayed in Table 7.

**Table 7:** Cardiovascular manifestations of dengue in the acute phase.

Reference	Study type, population size	Cardiac complication	Number of participants affected, n(%)
**Agudelo-Salas et al [21]** **2017**	Cross-sectional (n = 64)	Elevated creatin phosphokinase-MB	27 (42%)
**Datta et al [22]** **2019**	Prospective (n = 15)	LVEF 35–45%	4 (27%)
		Bradyarrhythmia	4 (27%)
		Pericardial effusion	2 (13%)
		Atrial fibrillation	1 (7%)
**Jayarajah et al [23]** **2018**	Prospective (n = 1,167)	Elevated troponin-I	2 (0.1%)
**Kularatne et al [24]** **2007**	Cross-sectional (n = 120)	Electrocardiogram: T inversion, ST depression, bundle branch block	75 (63%)
**La-Orkhun et al [25]** **2011**	Prospective (n = 35)	Electrocardiogram: AV-block	5 (14%)
**Lakshman et al [26]** **2018**	Prospective (n = 50)	LVEF < 50%	8 (16%)
		Elevated cardiac biomarkers (troponin-I, creatin phosphokinase-MB or myoglobin)	13 (26%)
		Electrocardiogram: QT prolongation	5 (10%)
		Electrocardiogram: Atrioventricular conduction defects	2 (4%)
**Li et al [27]** **2016**	Cross-sectional (n = 1,782)	Myocarditis	201 (11%)
		Undefined low LVEF	51 (3%)
**Mansanguan et al [28]** **2021**	Prospective (n = 81)	Elevated cardiac biomarkers	6 (7%)
		Left ventricular systolic dysfunction	3 (4%)
		Diastolic dysfunction	3 (4%)
		Pericardial effusion	6 (7%)
Miranda et al [29] 2013	Prospective (n = 81)	Elevated troponin-I	6 (7%)
		Elevated N-terminal pro brain natriuretic peptide	22 (27%)
		Myocarditis with cardiogenic shock	2 (2%)
**Obeyesekere et al [30]** **1973**	Case series (n = 35)	Myocarditis	24 (69%)
**Pothapregada et al [31]** **2016**	Retrospective (n = 254)	Pericardial effusion	3 (1%)
		Global hypokinesia	3 (1%)
		Diastolic dysfunction	2 (1%)
		Myocarditis	5 (2%)
**Satarasinghe et al [32]** **2007**	Prospective (n = 217)	Myocarditis	44 (20%)
**Sharda et al [33]** **2013**	Case series (n = 8)	LVEF < 45%	1 (13%)
**Sheetal et al [34]** **2016**	Cross-sectional (n = 100)	Sinus bradycardia	32 (32%)
		Myocarditis	0 (0%)
**Torres et al [35]** **2013**	Post-mortem (n = 44)	Myocarditis (histological)	8 (17%)
**Wali et al [36]** **1998**	Prospective (n = 17)	LVEF < 40%	7 (41%)
		Global hypokinesia	12 (71%)
		Electrocardiogram: ST/T changes	5 (29%)
**Weerakoon et al [37]** **2011**	Prospective (n = 166)	Myocarditis	45 (27%)
**Yadav et al [38]** **2013**	Prospective (n = 67)	Myocarditis	32 (48%)
		LVEF <50%	28 (42%)

LVEF: Left ventricular ejection fraction.

Cardiovascular involvement has been reported in several other viral and parasitic diseases common to the Amazon Basin. These involve different Arboviruses (Chikungunya, Yellow Fever, Zika), Leishmaniasis and malaria (Table 8). The fact that so many diseases, caused by different infectious pathogens, can all affect the myocardium, may suggest a common pathway to myocardial involvement. However, such a mechanism is not well described in studies of the above-mentioned neglected tropical diseases.

**Table 8:** Cardiac involvement in other tropical infections.

Reference	Study type, population size	Cardiac manifestation	Affected participants, n(%)
**Chikungunya**			
**Mendoza et al [39]** **2015**	Prospective (n = 83)	Rhythm disturbance	43 (52%)
		Sudden cardiac death	2 (2%)
**Gonzalez Carta et al [40]** **2018**	Prospective (n = 280)	Rhythm disturbance	126 (45%)
		Atrial fibrillation	18 (6%)
**Villamil-Gómez et al [41]** **2016**	Case series (n = 42)	Myocarditis	30 (71%)
**Koeltz et al [42]** **2018**	Prospective (n = 64)	Myocarditis	2 (3%)
**Yellow fever**			
**Paixão et al [43]** **2019**	Prospective (n = 70)	Left ventricular dysfunction	4 (6%)
		Myocarditis	1 (1%)
		Elevated troponin I	0 (0%)
		Abnormal electrocardiogram	36 (52%)
**Zika**			
**Gonzalez Carta et al [44]** **2017**	Case series (n = 9)	Arrhythmia	8 (89%)
		Low LVEF	5 (56%)
		Pericardial effusion	1 (11%)
**Leishmaniasis**			
**Dionisio et al [45]** **2011**	Retrospective (n = 51)	Myocarditis with acute heart failure	1 (2%)
**Malaria**			
**Nayak et al [46]** **2013**	Prospective (n = 100)	Elevated cardiac biomarkers	14 (14%)
		Cardiac dysfunction by echocardiography	17 (17%)
**Günther et al [47]** **2003**	Cross-sectional (n = 161)	Elevated troponin T	1 (0.6%)
		Abnormal electrocardiogram	23 (14%)
**Mohapatra et al [48]** **2000**	Cross-sectional (n = 195)	Elevated troponin T / CK-MB	7 (13%)
		Abnormal electrocardiogram	12 (6%)
**Ehrhardt et al [49]** **2005**	Cross-sectional (n = 400)	Elevated NT-proBNP	226 (57%)
		Elevated CK-MB	62 (16%)

CK-MB = creatine phosphokinase-MB, LVEF = Left ventricular ejection fraction, NT-proBNP = N-terminal proB natriuretic peptide.

**Fig 4:**
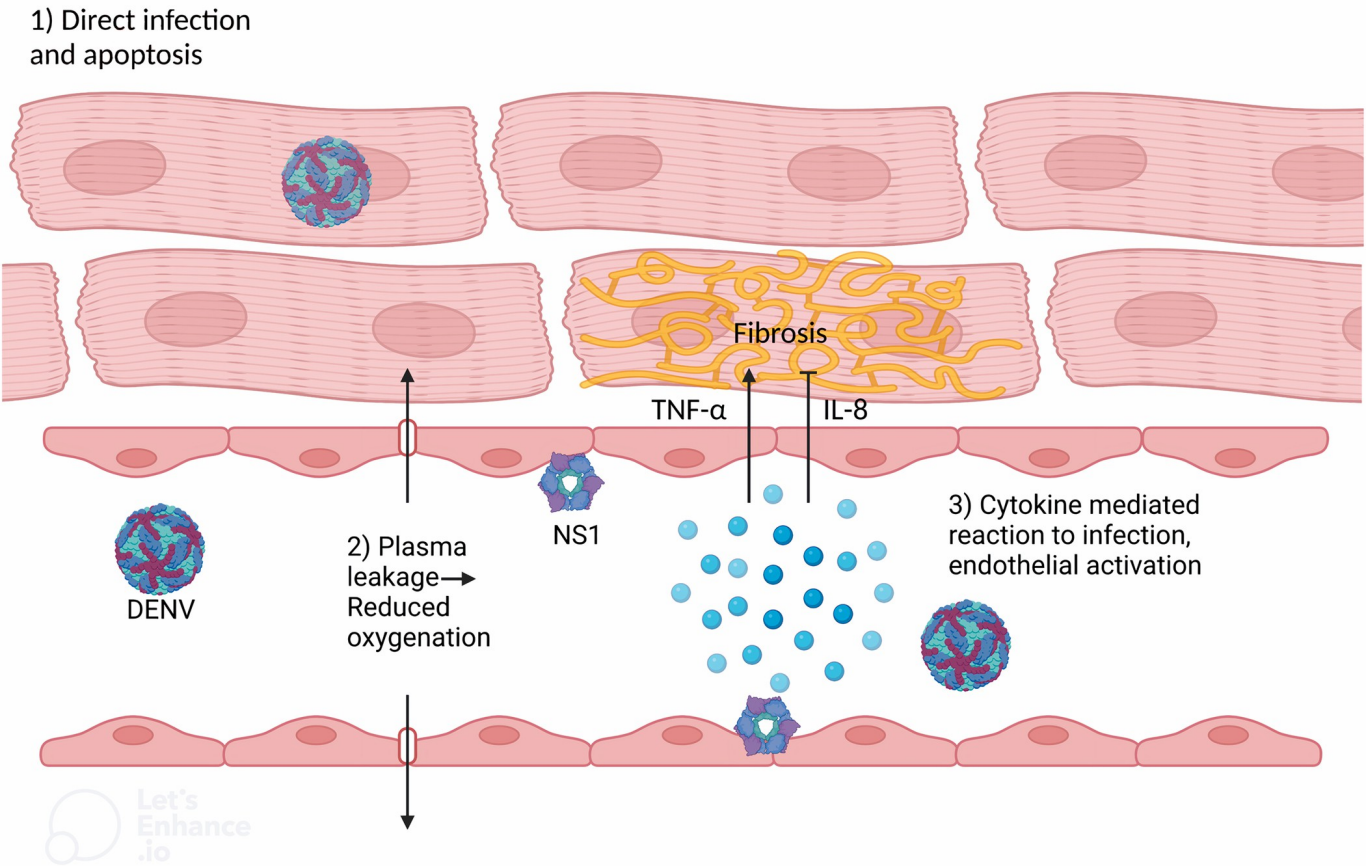
Proposed mechanisms of cardiac injury in dengue. Potential mechanisms for dengue induced myocardial impairment: (1) Direct infection of cardiomyocytes causing apoptosis and inflammation. (2) Vascular leakage induced by NS1, which leads to intravascular volume depletion and reduced preload, eventually causing cardiac ischemia. NS1 has also been associated with endothelial dysfunction and altered coronary microcirculation. (3) Inflammation in the cardiac vasculature and myocardial interstitial edema driven by an imbalanced immune response with release of pro-inflammatory cytokines. Over time the proposed mechanisms may lead to myocardial fibrosis and impaired contractility.

While volume depletion caused by vascular leakage can lead to organ dysfunction, and consequently affect the heart, other mechanisms are relatively unexplored. Proposed mechanisms between dengue and myocardial injury involve endothelial dysfunction [50], an imbalanced immune response to the virus [51], or dengue induced apoptosis [52] (Fig 4). Studies have examined cytokine expression in dengue fever and attempted to link this to organ specific complications. Expression of tumor necrosis factor alpha (TNF-α) and interleukin 8 (IL-8), among others, correlate with disease severity of dengue [53–55], and severe disease is associated with an excessive and long-lasting inflammatory response with inadequate response to anti-inflammatory cytokines [55]. Increased expression of TNF-α has been associated with decreased myocardial contractile function and worse outcome in heart failure participants [56], whereas higher levels of IL-8 are associated lower risk of myocardial ischemia [57]. Interestingly, the protective effect of IL-8 was only significant in women and not in men. This could indicate that IL-8 has some cardio-protective effect in women, and this could be related to the sex difference we observed in our study. However, the cytokines are not specific to dengue, and some studies showed no difference in TNF-α-expression in different severity grades. Cabrera-Rego et al found that cardiovascular complications (myocarditis, pericarditis) were more frequent in men with dengue compared to women (risk ratio of 1.94, P<0.001) [7]. Although the study did not report on comorbidities or assess cardiovascular risk factors, the findings indicate that men possibly could be more prone to cardiovascular complications in dengue. This finding is in line with our hypothesis and results.

Studies suggest that TNF-α inhibits downregulation of the inflammatory response during ischemia, and that ongoing inflammation leads to further damage of cardiomyocytes, increasing the long-term risk of heart failure [58]. Endothelial cells may be another activator of fibrosis, by release of cytokines and by endothelial to mesenchymal transformation, contributing directly to fibrosis [59]. Replication of dengue virus has been observed in myocardial fibers and in cardiac endothelial cells [51,60]. In accordance with this, we found that prior dengue was associated with lower LV systolic function, suggesting that cardiac involvement may persist beyond the acute phase of dengue.

As described by Tschöpe et al [61], viruses associated with myocarditis may be distinguished by how they affect the heart: direct infiltration (such as adenovirus and enteroviruses) and indirect by triggering an autoimmune mimicry or cytokine storm (such as influenza A and hepatitis C virus). Due to a limited number of studies, it is not possible categorize dengue, nor determine whether it causes myocardial impairment by a mix of these mechanisms. An overall barrier is the limited ability to translate animal experimental models of dengue to humans [2]. From a hypothesis generating perspective, studies have also shown increased risk of myocardial infarction in participants with myocarditis, proposing that myocarditis potentially may accelerate atherosclerosis [62,63]. Moreover, it remains unclear why some participants fully recover without residual myocardial damage following viral myocarditis, whereas others experience long-term injury [61]; something which could represent a key explanatory variable for our findings.

An increasing number of prior episodes of dengue was associated with declining LV systolic function in men, indicating a dose-response relationship. Pathogen burden has previously been associated with morbidity and mortality in other cardiovascular diseases [64,65], and this could also be the case for dengue and cardiac function. Furthermore, there is an overlap in populations experiencing non-communicable diseases and neglected tropical diseases, termed “the poorest of the rich” [66,67]. A study showed that individuals with diabetes and hypertension had higher risk of severe dengue [68]. The state of Acre is among the poorer regions of Brazil [69], and Brazil is home to considerable inequality as measured by the Gini coefficient [70]. Although consensus is lacking, dengue fever is generally considered to be a disease of poverty [71]. In this study, a history of clinical dengue was associated with living in urban areas and female sex, but no relationship was found with income or education. These results may be confounded by the fact that people living closer to health care centers are more likely to seek medical care and get a diagnosis of dengue.

Because of our study design and relatively small, yet significant associations, our results should be considered hypothesis-generating and may offer a future perspective for assessing long-term LV function following DENV infection. The prevalence of cardiovascular disease is increasing in low- and middle-income countries; the same regions where dengue virus is widespread. If dengue virus is related to cardiovascular disease, it could be of paramount clinical value to elucidate such a relationship, paving the road for novel preventative strategies and targets to improve cardiovascular health. Considering this, echocardiography could on a hypothesis-generating basis be used as a tool for screening of LV dysfunction in participants with prior or ongoing dengue, thus improving diagnosis and facilitating risk stratification. For those with impairment, follow-up programs could be implemented to evaluate whether this persists beyond the acute setting, allowing for identification of participants with need for medical intervention. However, this must be consolidated in future studies and lack of resources in dengue endemic areas could represent a barrier to the use of echocardiography. Although the rate of ECG findings in general was low, we found that the number of ECG alterations, among participants with a history of dengue, was associated with more echocardiographic abnormalities. Despite this finding being based on a small number of participants, it could indicate that ECG, especially in resource limited areas and where echocardiography is not available, may serve as a gateway to identify cardiac disease following dengue infection.

The major limitation of this study is that it was a cross-sectional study based on a self-reported history of clinical DENV infection. As demonstrated by antibody analyses, self-reported dengue had moderate accuracy to predict positive antibody titers. This could be due to subclinical and mild infections, where diagnostic testing is not performed, or due to recall bias. Furthermore, antibody analyses are liable to cross-reactivity from other arboviruses such as Zika and Chikungunya virus, which are also widespread in this region. However, the amount of Zika cases has decreased drastically since the initial epidemic, and in 2019 there were 691,000 confirmed cases of dengue and 1,800 confirmed cases of Zika in Brazil [3]. Unfortunately, we had no access to conduct plaque reduction neutralization test to quantify levels of dengue neutralizing antibodies. We lacked of information on disease severity, which could be correlated with greater risk of cardiac sequelae, and lack of time since last dengue fever episode. A potential bias is that people who seek out medical care due to underlying cardiovascular disease, could be more willing to participate in this study. We did not analyze cardiac biomarkers such as troponins or creatin phosphokinase-MB, nor was it possible to perform cardiac magnetic resonance imaging for myocarditis, due to lack of infrastructure and equipment in this rural part of the Amazon Basin. Furthermore, we did not assess the wall motion score index in echocardiograms, although this could have contributed with additional information on myocardial function. Cardiovascular symptoms did not occur more frequently in those with altered LVEF and/or GLS. This may be because symptoms first occur with more advanced cardiac impairment, whereas most participants in this study presented with only modest impairment of LV contractility. The study suffers from lack of a control group located outside of Amazonas. A major challenge is that normal values of echocardiographic parameters may vary even within different regions of Brazil, which represents a source of bias for such a comparison. Because of our study design, we cannot delineate whether worsening in LV function was because of unmeasured confounding factors or reverse causation. However, we had a clear hypothesis prior to commencing on this study, we did not seek to make causal inferences, and sought to adjust our multivariable models for relevant confounders.

In conclusion, a self-reported history of clinical DENV infection was associated with lower myocardial function as measured by left ventricular ejection fraction, global longitudinal and circumferential strain in men from the Amazon Basin. No relationship was found when assessing the entire population or women. A history of DENV infection was associated with greater odds of living in urban areas and female sex.

## Supporting information

S1 Table Dengue incidence in Acre, 1990–2017. (DOCX)

S2 Table Variance inflation factor of sociodemographic factors. (DOCX)

S3 Table Baseline characteristics stratified by sex. (DOCX)

S4 Table Baseline characteristics by history of malaria. (DOCX)

S5 Table Baseline characteristics by history of malaria in men. (DOCX)

S6 Table Baseline characteristics by history of malaria in women. (DOCX)

S7 Table Baseline characteristics by history of dengue in women. (DOCX)

S8 Table Association between number of dengue episodes and LV systolic function. (DOCX)

S1 Appendix Supplemental methods. (DOCX)

S1 File (XML)
