# Supplementary material for: Potential role of transthoracic echocardiography for screening LV systolic dysfunction in patients with a history of dengue infection. A cross-sectional and cohort study and review of the literature
Source: PLoS One. 2022 Nov 18;17(11):e0276725. doi: 10.1371/journal.pone.0276725 (PMC9674131; doi:10.1371/journal.pone.0276725)
Supplement: S1 Table — (DOCX) [file pone.0276725.s001.docx]

## S1 Table

## Dengue incidence in Acre, 1990-2017

|  | Cases / 100,000 inhabitants |
| --- | --- |
| 1990-1999 | 0 |
| 2000 | 100-300 |
| 2001 | 300-500 |
| 2002 | 100-300 |
| 2003 | 100-300 |
| 2004 | >500 |
| 2005 | 300-500 |
| 2006 | 0.1-100 |
| 2007 | 0.1-100 |
| 2008 | 300-500 |
| 2009 | >500 |
| 2010 | >500 |
| 2011 | >500 |
| 2012 | 300-500 |
| 2013 | 300-500 |
| 2014 | >500 |
| 2015 | >500 |
| 2016 | 100-300 |
| 2017 | 100-300 |

Source: Ministério da Saúde. Monitoramento dos casos de arboviroses urbanas causados por vírus transmitidos por Aedes (dengue, chikungunya e zika), semanas epidemiológicas 1 a 53, 2020. Bol Epidemiológico. 2021;52: 1–31.
