# Supplementary material for: Potential role of transthoracic echocardiography for screening LV systolic dysfunction in patients with a history of dengue infection. A cross-sectional and cohort study and review of the literature
Source: PLoS One. 2022 Nov 18;17(11):e0276725. doi: 10.1371/journal.pone.0276725 (PMC9674131; doi:10.1371/journal.pone.0276725)
Supplement: S2 Table — (DOCX) [file pone.0276725.s002.docx]

## S2 Table

## Variance inflation factor of sociodemographic factors

|  | Variance inflation factor (VIF) | Tolerance |
| --- | --- | --- |
| Urban environment | 1.31 | 0.77 |
| Family income* | 1.53 | 0.65 |
| Body mass index | 1.07 | 0.94 |
| House type | 1.03 | 0.97 |
| Insecure job situation | 1.29 | 0.78 |
| Education | 1.30 | 0.77 |

*log-transformed
