# Supplementary material for: Potential role of transthoracic echocardiography for screening LV systolic dysfunction in patients with a history of dengue infection. A cross-sectional and cohort study and review of the literature
Source: PLoS One. 2022 Nov 18;17(11):e0276725. doi: 10.1371/journal.pone.0276725 (PMC9674131; doi:10.1371/journal.pone.0276725)
Supplement: S3 Table — (DOCX) [file pone.0276725.s003.docx]

| S3 TableBaseline characteristics stratified by sex | | | |
| --- | --- | --- | --- |
|  | **Male**  (n=205) | **Female**  (n=316) | **P** |
| **Baseline** |  |  |  |
| Age, years | 39 ± 14 | 40 ± 15 | 0.39 |
| Recent malaria infection, n(%) | 36 (18%) | 28 (9%) | 0.003 |
| BMI, kg/m^2^ | 26 ± 4 | 28 ± 6 | <0.001 |
| Present smoker, n(%) | 82 (40%) | 104 (33%) | 0.099 |
| Hypertension, n(%) | 77 (38%) | 108 (34%) | 0.43 |
| Hypercholesterolemia, n%) | 23 (11%) | 51 (16%) | 0.12 |
| Diabetes, n(%) | 8 (4%) | 19 (6%) | 0.29 |
| SBP, mmHg | 133 ± 16 | 130 ± 21 | 0.038 |
| Heart rate, bpm | 68 ± 12 | 77 ± 13 | <0.001 |
| Rheumatic heart disease, n(%) | 8 (4%) | 4 (1%) | 0.050 |
| History of COVID-19, n(%) | 18 (9%) | 28 (9%) | 0.97 |
| History of dengue, n(%) | 84 (41%) | 169 (54%) | 0.005 |
| Number of dengue infections |  |  |  |
| 0 | 121 (59%) | 147 (47%) | 0.041 |
| 1 | 55 (27%) | 110 (35%) |  |
| 2 | 17 (8%) | 44 (14%) |  |
| ≥3 | 12 (6%) | 15 (5%) |  |
|  |  |  |  |
| **Socioeconomic** |  |  |  |
| Family income in real, BRL | 1,500 [1000 to 2,800] | 1,200 [800 to 2,000] | 0.003 |
| Family income in Euros, € | 275 [183 to 513] | 220 [147 to 366] |  |
| Insecure job situation, n(%) | 110 (54%) | 194 (61%) | 0.080 |
| Education, n(%) |  |  | 0.70 |
| No formal education | 15 (7%) | 16 (5%) |  |
| Primary school | 73 (36%) | 108 (34%) |  |
| Secondary school | 82 (40%) | 135 (43%) |  |
| Higher academic | 35 (17%) | 57 (18%) |  |
| Urban living area, n(%) | 89 (43%) | 171 (54%) | 0.017 |
| House type, n(%) |  |  | 0.22 |
| Wood | 146 (71%) | 209 (66%) |  |
| Brick | 59 (29%) | 107 (34%) |  |
| Use of mosquito bed net, n(%) | 101 (49%) | 181 (57%) | 0.073 |
| Use of mosquito repellent, n(%) | 12 (6%) | 28 (9%) | 0.21 |
|  |  |  |  |
| **Biochemistry** |  |  |  |
| CRP, mg/dL | 0.0 (0.0 to 0.0) | 0.0 (0.0 to 0.0) | 0.43 |
| Hemoglobin, g/dL | 15.2 ± 1 | 13.4 ± 1 | <0.001 |
| Leukocytes, mm^3^ | 5920 [4940 to 7000] | 6475 [5350 to 7870] | <0.001 |
| Reticulocytes, % | 0.8 [0.6 to 0.9] | 0.7 [0.6 to 0.9] | 0.009 |
| Platelets, mm^3^ | 219 ± 81 | 245 ± 61 | <0.001 |
| Creatinine, mg/dL | 0.9 [0.8 to 1.1] | 0.7 [0.6 to0.9] | <0.001 |
| Bilirubin total, mg/dL | 0.4 [0.3 to 0.6] | 0.3 [0.2 to 0.4] | <0.001 |
| INR | 1.03 ± 0.9 | 1.00 ± 0.11 | 0.002 |
| Blood glucose, mg/dL | 94 [86 to 111] | 96 [87 to 112] | 0.26 |
|  |  |  |  |
| **Electrocardiogram** |  |  |  |
| Left ventricular hypertrophy, n(%) | 24 (12%) | 6 (2%) | <0.001 |
| Left bundle branch block, n(%) | 1 (1%) | 0 (0%) | 0.30 |
| Right bundle branch block, n(%) | 1 (0.5%) | 1 (<3%) | 0.76 |
| Pathological Q-waves, n(%) | 5 (2%) | 4 (1%) | 0.32 |
|  |  |  |  |
| **Echocardiography** |  |  |  |
| LV ejection fraction, % | 56 ± 6 | 58 ± 5 | <0.001 |
| LVEF<50%, n(%) | 26 (13%) | 11 (4%) | <0.001 |
| GLS, % | -18.6 ± 2 | -20.0 ± 2 | <0.001 |
| GCS, % | -20.2 ± 4 | -21.5 ± 4 | <0.001 |
| GLS>-16%, n(%) | 24 (12%) | 2 (1%) | <0.001 |
| Number of hypokinetic segments | 4 (2 to 6) | 2 (1 to 4) | <0.001 |
| LV mass index, g/m^2^ | 76 ± 16 | 63 ± 14 | <0.001 |
| LAVI, mL/m^2^ | 20 ± 5 | 18 ± 4 | <0.001 |
| LAVI>34 mL/m², n(%) | 4 (2%) | 8 (3%) | 0.67 |
| e’, cm/s | 13.2 ± 4 | 13.0 ± 4 | 0.50 |
| Lateral e’<10 cm/s, n(%) | 29 (14%) | 45 (14%) | 0.98 |
| Septal e’<7 cm/s, n(%) | 17 (8%) | 30 (10%) | 0.64 |
| E/e’>14, n(%) | 8 (4%) | 16 (5%) | 0.54 |
| E/A-ratio | 1.4 ± 0.5 | 1.3 ± 0.4 | 0.13 |
| TAPSE, mm | 2.1 ± 0.3 | 2.0 ± 0.3 | 0.044 |
| Tricuspid regurgitation >3.8 m/s, n(%) | 1 (1%) | 0 (0%) | 0.21 |
| BMI = body mass index, BRL = Brazilian real (local currency), ECG = electrocardiogram, GCS = global circumferential strain, GLS = global longitudinal strain, LAVI = left atrial volume index, LV = left ventricular, LVEF = left ventricular ejection fraction, LVMI = left ventricular mass index, SBP = systolic blood pressure, TAPSE = Tricuspid annular plane systolic excursion | | | |
|  | | | |
