# Supplementary material for: Potential role of transthoracic echocardiography for screening LV systolic dysfunction in patients with a history of dengue infection. A cross-sectional and cohort study and review of the literature
Source: PLoS One. 2022 Nov 18;17(11):e0276725. doi: 10.1371/journal.pone.0276725 (PMC9674131; doi:10.1371/journal.pone.0276725)
Supplement: S4 Table — (DOCX) [file pone.0276725.s004.docx]

| S4 TableBaseline characteristics stratified by history of malaria | | | |
| --- | --- | --- | --- |
|  | **No history of malaria** (n=203) | **History of malaria** (n=318) | **P** |
|  |  |  |  |
| **Baseline** |  |  |  |
| Age, years | 38 ± 15 | 40 ± 15 | 0.15 |
| Men, n(%) | 55 (27%) | 150 (47%) | <0.001 |
| BMI, kg/m^2^ | 27 ± 5 | 27 ± 5 | 0.16 |
| Present smoker, n(%) | 49 (24%) | 137 (43%) | <0001 |
| Hypertension, n(%) | 75 (37%) | 110 (35%) | 0.58 |
| Hypercholesterolemia, n%) | 28 (14%) | 46 (15%) | 0.83 |
| Diabetes, n(%) | 10 (5%) | 17 (5%) | 0.83 |
| SBP, mmHg | 133 ± 20 | 130 ± 18 | 0.16 |
| Heart rate, bpm | 76 ± 14 | 72 ± 12 | <0.001 |
| Rheumatic heart disease, n(%) | 5 (3%) | 7 (2%) | 0.85 |
| History of COVID-19, n(%) | 26 (13%) | 20 (6%) | 0.011 |
| History of dengue, n(%) | 129 (64%) | 124 (39%) | <0.001 |
| Number of dengue episodes |  |  | <0.001 |
| 1 | 83 (41%) | 82 (26%) |  |
| 2 | 30 (15%) | 31 (10%) |  |
| 3-4 | 16 (8%) | 11 (4%) |  |
|  |  |  |  |
| **Biochemistry** |  |  |  |
| CRP, mg/dL | 0.0 (0.0 to 0.0) | 0.0 (0.0 to 0.0) | 0.41 |
| Hemoglobin, g/dL | 13.9 ± 1.3 | 14.2 ± 1.3 | 0.006 |
| Leukocytes, /mm^3^ | 6320 (5140 to 7590) | 6165 (5130 to 7420) | 0.74 |
| Reticulocytes, % | 0.8 (0.6 to 0.9) | 0.8 (0.6 to 0.9) | 0.78 |
| Platelets, /mm^3^ | 244 ± 61 | 229 ± 75 | 0.020 |
| Creatinine, mg/dL | 0.9 (0.7 to 1.0) | 0.8 (0.7 to 1.0) | 0.064 |
| Bilirubin total, mg/dL | 0.3 (0.2 to 0.5) | 0.4 (0.2 to 0.5) | 0.11 |
| INR | 1.0 ± 0.1 | 1.0 ± 0.1 | 0.87 |
| Blood glucose, mg/dL | 93 (86 to 109) | 97 (87 to 113) | 0.088 |
|  |  |  |  |
| **Electrocardiogram** |  |  |  |
| Left ventricular hypertrophy, n(%) | 3 (2%) | 27 (9%) | <0.001 |
| Left bundle branch block, n(%) | 0 (0%) | 1 (1%) | 0.55 |
| Right bundle branch block, n(%) | 2 (3%) | 0 (0%) | 0.017 |
| Pathological Q-waves, n(%) | 1 (1%) | 8 (4%) | 0.30 |
|  |  |  |  |
| **Echocardiography** |  |  |  |
| LV ejection fraction, % | 58 ± 5 | 57 ± 5 | 0.34 |
| LVEF<50%, n(%) | 14 (7%) | 23 (7%) | 0.88 |
| GLS, % | -19.5 ± 2 | -19.4 ± 2 | 0.63 |
| GCS, % | -20.6 ± 4 | -21.2 ± 4 | 0.18 |
| GLS>-16%, n(%) | 6 (3%) | 20 (6%) | 0.088 |
| LV mass index, g/m^2^ | 65.3 ± 16 | 70.0 ± 16 | 0.001 |
| LAVI, mL/m^2^ | 18.1 ± 4 | 19.6 ± 5 | <0.001 |
| LAVI>34 mL/m², n(%) | 2 (1%) | 0 (0%) | 0.076 |
| e’, cm/s | 13.2 ± 4 | 13.0 ± 4 | 0.48 |
| Lateral e’<10 cm/s, n(%) | 23 (11%) | 51 (16%) | 0.13 |
| Septal e’<7 cm/s, n(%) | 18 (9%) | 29 (9%) | 0.92 |
| E/e’>14, n(%) | 2 (1%) | 3 (1%) | 0.96 |
| E/A-ratio | 1.3 ± 0.4 | 1.3 ± 0.4 | 0.26 |
| TAPSE, mm | 2.0 ± 0.3 | 2.0 ± 0.3 | 0.43 |
| Tricuspid regurgitation >3.8 m/s, n(%) | 1 (1%) | 0 (0%) | 0.21 |
| BMI = body mass index, GCS = global circumferential strain, GLS = global longitudinal strain, LAVI = left atrial volume index, LV = left ventricular, LVEF = left ventricular ejection fraction, LVMI = left ventricular mass index, SBP = systolic blood pressure, TAPSE = Tricuspid annular plane systolic excursion | | | |
