# Supplementary material for: Potential role of transthoracic echocardiography for screening LV systolic dysfunction in patients with a history of dengue infection. A cross-sectional and cohort study and review of the literature
Source: PLoS One. 2022 Nov 18;17(11):e0276725. doi: 10.1371/journal.pone.0276725 (PMC9674131; doi:10.1371/journal.pone.0276725)
Supplement: S5 Table — (DOCX) [file pone.0276725.s005.docx]

| S5 TableBaseline characteristics stratified by history of malaria in men | | | |  |
| --- | --- | --- | --- | --- |
|  | **No history of malaria** (n=55) | **History of malaria** (n=150) | **P** | |
| **Baseline** |  |  |  | |
| Age, years | 34 ± 14 | 40 ± 14 | 0.004 | |
| BMI, kg/m^2^ | 26 ± 4 | 26 ± 4 | 0.69 | |
| Present smoker, n(%) | 11 (20%) | 71 (47%) | <0.001 | |
| Hypertension, n(%) | 24 (44%) | 53 (35%) | 0.28 | |
| Hypercholesterolemia, n%) | 6 (11%) | 17 (11%) | 0.93 | |
| Diabetes, n(%) | 1 (2%) | 7 (5%) | 0.35 | |
| SBP, mmHg | 136 ± 17 | 132 ± 15 | 0.087 | |
| Heart rate, bpm | 72 ± 14 | 67 ± 11 | 0.003 | |
| Rheumatic heart disease, n(%) | 2 (4%) | 6 (4%) | 0.91 | |
| History of COVID-19, n(%) | 7 (13%) | 11 (7%) | 0.23 | |
| History of dengue, n(%) | 30 (55%) | 54 (36%) | 0.017 | |
| Number of dengue episodes |  |  | 0.19 | |
| 1 | 20 (36%) | 35 (23%) |  | |
| 2 | 6 (11%) | 11 (7%) |  | |
| 3-4 | 4 (7%) | 8 (5%) |  | |
|  |  |  |  | |
| **Biochemistry** |  |  |  | |
| CRP, mg/dL | 0.0 (0.0 to 0.0) | 0.0 (0.0 to 0.0) | 0.075 | |
| Hemoglobin, g/dL | 15.3 ± 1.1 | 15.2 ± 1.0 | 0.23 | |
| Leukocytes, /mm^3^ | 6280 (4970 to 7360) | 5830 (4940 to 6820) | 0.18 | |
| Reticulocytes, % | 0.8 (0.7 to 0.9) | 0.8 (0.6 to 0.9) | 0.53 | |
| Platelets, /mm^3^ | 234 ± 68 | 213 ± 84 | 0.097 | |
| Creatinine, mg/dL | 1.0 (0.9 to 1.1) | 0.9 (0.8 to 1.1) | 0.085 | |
| Bilirubin total, mg/dL | 0.4 (0.3 to 0.6) | 0.4 (0.3 to 0.5) | 0.47 | |
| INR | 1.0 ± 0.1 | 1.0 ± 0.1 | 0.36 | |
| Blood glucose, mg/dL | 93 (82 to 106) | 95 (86 to 112) | 0.21 | |
|  |  |  |  | |
| **Electrocardiogram** |  |  |  | |
| Left ventricular hypertrophy, n(%) | 2 (4%) | 22 (15%) | 0.030 | |
| Left bundle branch block, n(%) | 0 (0%) | 1 (1%) | 0.64 | |
| Right bundle branch block, n(%) | 1 (4%) | 0 (0%) | 0.031 | |
| Pathological Q-waves, n(%) | 0 (0%) | 5 (4%) | 0.29 | |
|  |  |  |  | |
| **Echocardiography** |  |  |  | |
| LV ejection fraction, % | 56 ± 6 | 56 ± 6 | 0.82 | |
| LVEF<50%, n(%) | 8 (15%) | 18 (12%) | 0.63 | |
| GLS, % | -18.5 ± 2 | -18.7 ± 2 | 0.65 | |
| GCS, % | -20.4 ± 4 | -20.2 ± 4 | 0.18 | |
| GLS>-16%, n(%) | 6 (11%) | 18 (12%) | 0.83 | |
| LV mass index, g/m^2^ | 71.6 ± 17 | 77.5 ± 15 | 0.017 | |
| LAVI, mL/m^2^ | 19.0 ± 5 | 20.8 ± 5 | 0.019 | |
| LAVI>34 mL/m², n(%) | 2 (4%) | 0 (0%) | 0.019 | |
| e’, cm/s | 14.1 ± 4 | 12.9 ± 4 | 0.026 | |
| Lateral e’<10 cm/s, n(%) | 4 (7%) | 25 (17%) | 0.087 | |
| Septal e’<7 cm/s, n(%) | 2 (4%) | 15 (10%) | 0.14 | |
| E/e’>14, n(%) | 0 (0%) | 1 (1%) | 0.54 | |
| E/A-ratio | 1.4 ± 0.5 | 1.3 ± 0.5 | 0.096 | |
| TAPSE, mm | 2.1 ± 0.3 | 2.1 ± 0.3 | 0.74 | |
| Tricuspid regurgitation >3.8 m/s, n(%) | 1 (2%) | 0 (0%) | 0.98 | |
| BMI = body mass index, GCS = global circumferential strain, GLS = global longitudinal strain, LAVI = left atrial volume index, LV = left ventricular, LVEF = left ventricular ejection fraction, LVMI = left ventricular mass index, SBP = systolic blood pressure, TAPSE = Tricuspid annular plane systolic excursion | | | | |
