# Supplementary material for: Potential role of transthoracic echocardiography for screening LV systolic dysfunction in patients with a history of dengue infection. A cross-sectional and cohort study and review of the literature
Source: PLoS One. 2022 Nov 18;17(11):e0276725. doi: 10.1371/journal.pone.0276725 (PMC9674131; doi:10.1371/journal.pone.0276725)
Supplement: S6 Table — (DOCX) [file pone.0276725.s006.docx]

| S6 TableBaseline characteristics stratified by history of malaria in women | | | |  |
| --- | --- | --- | --- | --- |
|  | **No history of malaria** (n=148) | **History of malaria** (n=168) | **P** | |
| **Baseline** |  |  |  | |
| Age, years | 40 ± 15 | 40 ± 15 | 1.00 | |
| BMI, kg/m^2^ | 28 ± 6 | 27 ± 5 | 0.60 | |
| Present smoker, n(%) | 38 (26%) | 66 (39%) | 0.010 | |
| Hypertension, n(%) | 51 (35%) | 57 (34%) | 0.92 | |
| Hypercholesterolemia, n%) | 22 (15%) | 29 (17%) | 0.56 | |
| Diabetes, n(%) | 9 (6%) | 10 (6%) | 0.96 | |
| SBP, mmHg | 131 ± 21 | 128 ± 20 | 0.25 | |
| Heart rate, bpm | 78 ± 13 | 77 ± 12 | 0.34 | |
| Rheumatic heart disease, n(%) | 3 (2%) | 1 (1%) | 0.26 | |
| History of COVID-19, n(%) | 19 (13%) | 9 (5%) | 0.020 | |
| History of dengue, n(%) | 99 (67%) | 70 (42%) | <0.001 | |
| Number of dengue episodes |  |  | <0.001 | |
| 1 | 63 (43%) | 47 (28%) |  | |
| 2 | 24 (16%) | 20 (12%) |  | |
| 3-4 | 12 (8%) | 3 (2%) |  | |
|  |  |  |  | |
| **Biochemistry** |  |  |  | |
| CRP, mg/dL | 0.0 (0.0 to 0.0) | 0.0 (0.0 to 0.0) | 0.90 | |
| Hemoglobin, g/dL | 13.4 ± 1.0 | 13.4 ± 1.0 | 0.60 | |
| Leukocytes, /mm^3^ | 6365 (5240 to 7720) | 6600 (5485 to 8065) | 0.14 | |
| Reticulocytes, % | 0.7 (0.6 to 0.9) | 0.7 (0.6 to 0.9) | 0.84 | |
| Platelets, /mm^3^ | 248 ± 59 | 243 ± 63 | 0.55 | |
| Creatinine, mg/dL | 0.8 (0.7 to 1.0) | 0.7 (0.6 to 0.9) | <0.001 | |
| Bilirubin total, mg/dL | 0.3 (0.2 to 0.4) | 0.3 (0.2 to 0.5) | 0.30 | |
| INR | 1.0 ± 0.1 | 1.0 ± 0.1 | 0.15 | |
| Blood glucose, mg/dL | 93 (86 to 110) | 98 (87 to 114) | 0.12 | |
|  |  |  |  | |
| **Electrocardiogram** |  |  |  | |
| Left ventricular hypertrophy, n(%) | 1 (1%) | 5 (3%) | 0.13 | |
| Left bundle branch block, n(%) | 0 (0% | 0 (0%) | NA | |
| Right bundle branch block, n(%) | 1 (2%) | 0 (0%) | 0.16 | |
| Pathological Q-waves, n(%) | 1 (2%) | 3 (3%) | 0.71 | |
|  |  |  |  | |
| **Echocardiography** |  |  |  | |
| LV ejection fraction, % | 58 ± 4 | 58 ± 5 | 0.81 | |
| LVEF<50%, n(%) | 6 (4%) | 5 (3%) | 0.60 | |
| GLS, % | -19.9 ± 2 | -20.1 ± 2 | 0.31 | |
| GCS, % | -20.8 ± 4 | -22.1 ± 4 | 0.007 | |
| GLS>-16%, n(%) | 0 (0%) | 2 (1%) | 0.18 | |
| LV mass index, g/m^2^ | 62.9 ± 15 | 63.3 ± 13 | 0.80 | |
| LAVI, mL/m^2^ | 17.8 ± 4 | 18.5 ± 4 | 0.15 | |
| LAVI>34 mL/m², n(%) | 0 (0%) | 0 (0%) | 1.00 | |
| e’, cm/s | 12.9 ± 4 | 13.1 ± 4 | 0.64 | |
| Lateral e’<10 cm/s, n(%) | 19 (13%) | 26 (16%) | 0.50 | |
| Septal e’<7 cm/s, n(%) | 16 (11%) | 14 (8%) | 0.45 | |
| E/e’>14, n(%) | 2 (1%) | 2 (1%) | 0.90 | |
| E/A-ratio | 1.3 ± 0.4 | 1.3 ± 0.4 | 0.59 | |
| TAPSE, mm | 2.0 ± 0.3 | 2.0 ± 0.3 | 0.84 | |
| Tricuspid regurgitation >3.8 m/s, n(%) | 0 (0%) | 0 (0%) | 1.00 | |
| BMI = body mass index, GCS = global circumferential strain, GLS = global longitudinal strain, LAVI = left atrial volume index, LV = left ventricular, LVEF = left ventricular ejection fraction, LVMI = left ventricular mass index, SBP = systolic blood pressure, TAPSE = Tricuspid annular plane systolic excursion | | | | |
