# Supplementary material for: Potential role of transthoracic echocardiography for screening LV systolic dysfunction in patients with a history of dengue infection. A cross-sectional and cohort study and review of the literature
Source: PLoS One. 2022 Nov 18;17(11):e0276725. doi: 10.1371/journal.pone.0276725 (PMC9674131; doi:10.1371/journal.pone.0276725)
Supplement: S7 Table — (DOCX) [file pone.0276725.s007.docx]

| S7 TableBaseline characteristics by history of dengue in women | | | | |
| --- | --- | --- | --- | --- |
|  | **No dengue**  (n=147) | **Dengue**  (n=169) | **P** |  |
| **Baseline** |  |  |  |  |
| Age, years | 40 ± 15 | 40 ± 15 | 0.81 |  |
| Recent malaria infection, n(%) | 14 (10%) | 14 (8%) | 0.70 |  |
| BMI, kg/m^2^ | 27.5 ± 6 | 27.6 ± 6 | 0.87 |  |
| Present smoker, n(%) | 54 (37%) | 50 (30%) | 0.18 |  |
| Hypertension, n(%) | 48 (33%) | 60 (36%) | 0.59 |  |
| Hypercholesterolemia, n(%) | 24 (16%) | 27 (16%) | 0.93 |  |
| Diabetes, n(%) | 9 (6%) | 10 (6%) | 0.94 |  |
| SBP, mmHg | 129 ± 21 | 130 ± 21 | 0.78 |  |
| Heart rate, bpm | 77 ± 11 | 78 ± 14 | 0.47 |  |
| Rheumatic heart disease, n(%) | 3 (2%) | 1 (1%) | 0.25 |  |
| Suspected history of COVID-19, n(%) | 10 (7%) | 18 (11%) | 0.23 |  |
| Number of dengue infections |  |  | NA |  |
| 1 | NA | 110 (65%) |  |  |
| 2 | NA | 44 (26%) |  |  |
| ≥3 | NA | 15 (9%) |  |  |
|  |  |  |  |  |
| **Socioeconomic** |  |  |  |  |
| Family income in real, BRL | 1000 (600 to 2000) | 1500 (1000 to 2180) | 0.003 |  |
| Family income in euros, € | 183 [110 to 366] | 275 [183 to 399] |  |  |
| Insecure job situation, n(%) | 96 (65%) | 98 (58%) | 0.18 |  |
| Education, n(%) |  |  | 0.61 |  |
| No formal education | 8(5%) | 8 (5%) |  |  |
| Primary school | 53 (36%) | 55 (33%) |  |  |
| Secondary school | 64 (44%) | 71 (42%) |  |  |
| Higher academic | 22 (15%) | 35 (21%) |  |  |
| Urban living area, n(%) | 66 (45%) | 105 (62%) | 0.002 |  |
| House type, n(%) |  |  | 0.036 |  |
| Wood | 106 (72%) | 103 (61%) |  |  |
| Brick | 41 (28%) | 66 (39%) |  |  |
| Use of mosquito bed net, n(%) | 94 (64%) | 87 (52%) | 0.025 |  |
| Use of mosquito repellent, n(%) | 15 (10%) | 13 (8%) | 0.43 |  |
|  |  |  |  |  |
| **Biochemistry** |  |  |  |  |
| CRP, mg/dL | 0.0 (0.0 to 0.0) | 0.0 (0.0 to 0.0) | 0.99 |  |
| Hemoglobin, g/dL | 13.4 ± 1 | 13.4 ± 1 | 0.60 |  |
| Leukocytes, mm^3^ | 6520 (5360 to 7870) | 6445 (5345 to 7859) | 0.87 |  |
| Reticulocytes, % | 0.7 (0.6 to 0.9) | 0.7 (0.6 to 0.9) | 0.56 |  |
| Platelets, mm^3^ | 245 ± 58 | 246 ± 64 | 0.81 |  |
| Creatinine, mg/dL | 0.7 [0.6 to 0.9] | 0.8 [0.7 to 0.9] | 0.012 |  |
| Bilirubin total, mg/dL | 0.3 [0.2 to 0.4] | 0.3 [0.2 to 0.4] | 0.70 |  |
| INR | 1.01 ± 0.13 | 0.99 ± 0.10 | 0.17 |  |
| Blood glucose, mg/dL | 94 [86 to 110] | 97 [87 to 114] | 0.23 |  |
|  |  |  |  |  |
| **Electrocardiogram** |  |  |  |  |
| Left ventricular hypertrophy, n(%) | 4 (3%) | 2 (1%) | 0.32 |  |
| Left bundle branch block, n(%) | 0 (0%) | 0 (0%) |  |  |
| Right bundle branch block, n(%) | 0 (0%) | 1 (1%) | 0.35 |  |
| Pathological Q-waves, n(%) | 3 (2%) | 1 (1%) | 0.25 |  |
|  |  |  |  |  |
| **Echocardiography** |  |  |  |  |
| LV ejection fraction, % | 58.1 ± 4 | 58.3 ± 5 | 0.78 |  |
| LVEF<50%, n(%) | 4 (3%) | 7 (4%) | 0.49 |  |
| GLS, % | -20.1 ± 1.8 | -19.9 ± 1.9 | 0.21 |  |
| GCS, % | -21.6 ± 3.6 | -21.4 ± 4.3 | 0.62 |  |
| GLS>-16%, n(%) | 0 (0%) | 2 (1%) | 0.19 |  |
| Number of hypokinetic segments | 2 (1 to 4) | 2 (1 to 4) | 0.90 |  |
| LV mass index, g/m^2^ | 63 ± 14 | 63 ± 15 | 0.84 |  |
| LAVI, mL/m^2^ | 19 ± 4 | 18 ± 4 | 0.18 |  |
| LAVI>34 mL/m², n(%) | 4 (3%) | 4 (2%) | 0.84 |  |
| e’, cm/s | 12.7 ± 4 | 13.2 ± 4 | 0.23 |  |
| Lateral e’<10 cm/s, n(%) | 22 (15%) | 23 (14%) | 0.73 |  |
| Septal e’<7 cm/s, n(%) | 12 (8%) | 18 (11%) | 0.45 |  |
| E/e’>14, n(%) | 8 (5%) | 8 (5%) | 0.77 |  |
| E/A-ratio | 1.3 ± 0.4 | 1.3 ± 0.4 | 0.81 |  |
| TAPSE, mm | 2.0 ± 0.3 | 2.0 ± 0.3 | 0.89 |  |
| Tricuspid regurgitation >3.8 m/s, n(%) | 0 (0%) | 0 (0%) | 1.00 |  |
| BMI = body mass index, BRL = Brazilian real (local currency), GCS = global circumferential strain, GLS = global longitudinal strain, LAVI = left atrial volume index, LV = left ventricular, LVEF = left ventricular ejection fraction, LVMI = left ventricular mass index, SBP = systolic blood pressure, TAPSE = Tricuspid annular plane systolic excursion | | | | |
