# Supplementary material for: Potential role of transthoracic echocardiography for screening LV systolic dysfunction in patients with a history of dengue infection. A cross-sectional and cohort study and review of the literature
Source: PLoS One. 2022 Nov 18;17(11):e0276725. doi: 10.1371/journal.pone.0276725 (PMC9674131; doi:10.1371/journal.pone.0276725)
Supplement: S8 Table — (DOCX) [file pone.0276725.s008.docx]

## S8 Table

## Association between number of dengue infections and LV systolic function

Beta per one increase in number of dengue infections

|  | Entire cohort (n=524) | | Men (n=202) | | Women (n=322) | |
| --- | --- | --- | --- | --- | --- | --- |
|  | Beta (95%CI) | P | Beta (95%CI) | P | Beta (95%CI) | P |
| **Unadjusted** |  |  |  |  |  |  |
| GLS | 0.12 (-0.09 to 0.33) | 0.26 | 0.62 (0.34 to 0.89) | <0.001* | -0.15 (-0.37 to 0.07) | 0.18 |
| GCS | 0.33 (-0.08 to 0.74) | 0.12 | 0.93 (0.34 to 1.52) | 0.002* | -0.02 (-0.50 to 0.46) | 0.93 |
| LVEF | -0.16 (-0.64 to 0.32) | 0.52 | -1.17 (-1.85 to -0.49) | 0.001* | 0.43 (-0.13 to 0.98) | 0.13 |
| *Significant in multivariable models adjusted for age, systolic blood pressure, heart rate, creatinine, diabetes, smoking, BMI, recent malaria infection, urban living area and income.  GCS = global circumferential strain, GLS = global longitudinal strain, LAVI = left atrial volume index, LVEF = left ventricular ejection fraction, LVMI = left ventricular mass index, SBP = systolic blood pressure, TAPSE = Tricuspid annular plane systolic excursion | | | | | | |
