# Supplementary material for: Potential role of transthoracic echocardiography for screening LV systolic dysfunction in patients with a history of dengue infection. A cross-sectional and cohort study and review of the literature
Source: PLoS One. 2022 Nov 18;17(11):e0276725. doi: 10.1371/journal.pone.0276725 (PMC9674131; doi:10.1371/journal.pone.0276725)
Supplement: S1 Appendix — (DOCX) [file pone.0276725.s009.docx]

## S1 Appendix

## Supplemental Methods

From a finger we collected blood for point-of-care analysis of blood glucose and thick and thin blood slides. A physician history of diabetes or fasting blood glucose >126mg/dL were categorized as diabetes [2]. Hypertension was defined as a history of hypertension or intake of anti-hypertensive medication or systolic blood pressure ≥140 mmHg and/or diastolic blood pressure ≥90 mmHg. Hypercholesterolemia was defined by a physician diagnosis or intake of lipid-lowering medication. For patients who reported shortness of breath during physical activity, we applied the four categories of the New York Heart Association functional classification to assess potential symptoms of heart failure. Intermittent claudication was defined as self-reported lower extremity pain that appeared while walking for more than 2 minutes and disappeared when stopping. In analyses of electrocardiographic data, we assessed presence of LV hypertrophy according to criteria proposed by Sokolow-Lyon and Cornell [1]. LV hypertrophy was categorized as present if one of the two criteria were positive. Q-waves were considered pathological if present in V1-V3, the width exceeded 1mm, depth >2mm and the amplitude was >25% of QRS complex [2].

Standard giemsa stained thick and thin blood smears were analyzed by two locally trained microscopists to determine presence of *Plasmodium*. Peripheral blood samples were collected in tubes of citrate, serum-separator and EDTA and immediately cooled at 2-8^o^C. In a mobile laboratory, plasma citrate was separated by centrifugation (12 minutes, 3200 rpm) within 30 minutes and transferred to Eppendorf tubes, which were cooled.

Serum-separator tubes underwent centrifugation (10 min, 3000rpm), and serum was stored at -20^o^C. Laboratory analyses were conducted at Citolab and Centro de Diagnósticos, Cruzeiro do Sul, Acre, Brazil. EDTA blood was used for assessing blood components (NX-350, Sysmex, Japan; Citolab) and manual counting of reticulocytes (Citolab), while citrate plasma was used for coagulation (Coagmaster 2.0, Wama Diagnóstica, Brazil; Citolab). Serum was analyzed for creatinine and bilirubin (Cobas c111, Roche Diagnostics, Switzerland; Citolab and Centro de Diagnósticos).

## References

1. Schillaci G, Verdecchia P, Borgioni C, Ciucci A, Guerrieri M, Zampi I, et al. Improved electrocardiographic diagnosis of left ventricular hypertrophy. Am J Cardiol. 1994;74: 714–719. doi:10.1016/0002-9149(94)90316-6

2. Delewi R, IJff G, van de Hoef TP, Hirsch A, Robbers LF, Nijveldt R, et al. Pathological Q Waves in Myocardial Infarction in Patients Treated by Primary PCI. JACC Cardiovasc Imaging. 2013;6: 324–331. doi:10.1016/j.jcmg.2012.08.018
